# Predicting risk factors for Epstein-Barr virus reactivation using Bayesian network analysis: a population-based study of high-risk areas for nasopharyngeal cancer

**DOI:** 10.3389/fonc.2024.1369765

**Published:** 2025-01-21

**Authors:** Zhiwen Zeng, Kena Lin, Xueqi Li, Tong Li, Xiaoman Li, Jiayi Li, Zule Ning, Qinxian Liu, Shanghang Xie, Sumei Cao, Jinlin Du

**Affiliations:** ^1^ School of Public Health, Guangdong Medical University, Dongguan, Guangdong, China; ^2^ Department of Cancer Prevention, Sun Yat-sen University Cancer Center, Guangzhou, China; ^3^ School of Public Health, Sun Yat-sen University, Guangzhou, China; ^4^ State Key Laboratory of Oncology in South China, Collaborative Innovation Center for Cancer Medicine, and Guangdong Key Laboratory of Nasopharyngeal Carcinoma Diagnosis and Therapy, Sun Yat-Sen University Cancer Center, Guangzhou, China

**Keywords:** Bayesian network, EBV reactivation, model construction, nasopharyngeal carcinoma, logistic regression

## Abstract

**Background and objective:**

Nasopharyngeal carcinoma (NPC) is a rare disease in most parts of the world, but it is highly prevalent in South China. Epstein-Barr virus (EBV) is one of the major risk factors for NPC. Hence, understanding the factors associated with the reactivation of EBV from the latent stage is crucial for preventing NPC. This study aimed to investigate the risk factors for EBV reactivation associated with NPC in high-prevalence areas in China using a Bayesian network (BN) model combined with structural equation modeling tools.

**Methods:**

The baseline information for this study was derived from NPC screening data from a population-based prospective cohort in Sihui City, Guangdong Province, China. We divided the data into a training dataset and a test dataset. We then constructed an interaction networktionba BN prediction model to explore the risk factors for EBV reactivation, which was compared with a conventional logistic regression model.

**Results:**

A total of 12,579 participants were included in the analyses, with 1596 participant pairs finally included after the use of a nested case-control study. The results of multivariable logistic regression showed that only being older than 60 years (OR = 1.718, 95% CI = 1.273,2.322) and being a current smoker (OR = 1.477, 95% CI = 1.167 - 1.872) were the risk factors for EBV reactivation. The results of the model constructed using BN showed that age and smoking were directly associated with EBV reactivation. In contrast, sex, education level, tea drinking, cooking, and family history of cancer were indirectly associated with EBV reactivation. Further, we predicted the risk of EBV reactivation using Bayesian inference and visualized the BN inference. Model prediction performance was evaluated using the test dataset. The results showed that the BN model slightly outperformed the traditional logistic regression model in all metrics.

**Conclusions:**

BN not only reflects the complex interaction between factors but also visualizes the prediction results. It has a promising application potential in the risk prediction of EBV reactivation associated with NPC.

## 1 Introduction

Nasopharyngeal carcinoma (NPC) is a rarely diagnosed disease in most parts of the world, with an age-standardized incidence rate usually lower than 1 case per 100,000 person-years. However, the rates are high in South China, South-East Asia, the Arctic, North Africa, and the Middle East among indigenous groups (1). Research has shown that the 5-year survival rate of stage I NPC in high-prevalence areas is as high as ≥85%. However, the 5-year survival rate of stage IV patients is only 20%, and the 5-year survival rate after distant metastasis is less than 5% (2). The proportion of early-stage patients among the patients attending the clinic is less than 30%. Most patients are already in advanced stages at the time of consultation because of the hidden location of the nasopharyngeal cavity and the lack of specificity of the early symptoms of NPC (3). Therefore, the early detection, diagnosis, and treatment of NPC are now central to its prevention and treatment. The Epstein-Barr virus (EBV) is a ubiquitous B-lymphotropic virus carried latently by almost all humans. It is usually first contracted in childhood, during which it either causes no symptoms or only mild ones (4). The International Agency for Research on Cancer has shown that EBV is an established cause of several human malignancies, including nasopharyngeal cancer (5). Approximately 95% of the global population is asymptomatically infected with EBV throughout their lives. However, EBV can be periodically reactivated in response to endogenous and environmental stresses (6, 7). During the transition of EBV from a latent infection state to a lytic replication phase, several latent and lytic gene products are expressed, contributing to epithelial cell genetic damage, immune system perturbation, and angiogenesis in the nasopharynx, thereby increasing the risk of nasopharyngeal carcinoma (8). Our previous studies have demonstrated that serum immunoglobulin A (IgA) antibodies against EBV nuclear antigen 1 (EBNA1/IgA) and viral capsid antigen (VCA/IgA) serve as serological biomarkers of EBV activation and are effective in predicting nasopharyngeal cancer (9, 10). Thus, understanding the risk factors for EBV reactivation associated with NPC is essential for preventing nasopharyngeal cancer.

It was previously found through the construction of logistic models that smoking (11), using solid fuel (12), and consuming salty food (12) were associated with EBV reactivation. However, the conventional logistic regression models fail to capture the complex network of interactions between multiple risk factors. Also, the assumption of linear additivity of the model may limit its use, leading to reduced efficacy of regression model tests and regression model failure (13–15). Furthermore, the model cannot identify direct or indirect risk factors. Accordingly, screening variables or constructing models based on the network structures is significant for analyzing the risk factors for EBV reactivation associated with NPC.

Bayesian networks (BNs), which were proposed by Judea Pearl (16), can better compensate for the shortcomings of logistic regression models. BNs comprise two components: a directed acyclic graph (DAG) that reflects the complex network of interactions among risk factors and a conditional probability table that depicts the correlation between variables (17, 18). The BN overcomes the correlation restrictions of the traditional regression models (i.e., the assumption of independence between variables), and it can also infer the probability of an unknown node when the node is known. An increasing number of studies have applied BN for predicting risk factors for diseases such as cardiovascular disease (19), stroke (20), and colorectal cancer prognosis (21). However, reports regarding the application of the BN in NPC studies, especially in the context of China, are limited. In addition, no BN modeling studies related to predicting the risk of EBV reactivation have been found. Therefore, considering the problems of the traditional prediction model, we attempted to construct the EBV reactivation prediction model based on the interactive network system and explore the predictive effect of the model, which is of great significance to public health.

Unbalanced datasets can lead to degraded model performance, so balancing the classes of EBV reactivation states is crucial for constructing BN models (22). Also, we used a nested case–control study approach and processed the data by matching 1:1 for several baseline characteristics and ill-defined variables to make the data more comparable and control for confounding bias as much as possible. Besides, in BN structure learning, we applied structural equation modeling tools, enhancing the stability of the constructed structures (23). In this study, we combined screening data from a high-prevalence area of NPC in South China, aiming to explore the risk factors for EBV reactivation by constructing a structural equation modeling (SEM)-treated BN model using a nested case–control study. The purpose was to intervene and reduce the risk of NPC in an early stage and provide a new idea for the prevention and treatment of NPC.

## 2 Materials and methods

### 2.1 Data sources and study participants

The baseline information for this study was obtained from a population-based prospective cohort study (South China Chronic Disease Cohort) in Sihui City, Guangdong Province, designed to systematically investigate the risk factors for common noncommunicable diseases in the local population. A total of 12,619 native permanent residents (defined as having lived in Sihui City for at least 6 months) aged 18 years or older were recruited between October 2017 and March 2021 to participate in the baseline study. The study was approved by the Human Ethics Committee of the Sun Yat-sen University Cancer Control Centre (SYSUCC), and written informed consent was obtained from all the participants.

Each participant was required to complete a structured questionnaire after being interviewed by a trained investigator. The questionnaire included basic demographic characteristics (sex, age, education level, marital status, income, etc.), general health status (personal and family history of communicable and noncommunicable diseases, family history of cancer, history of drug use, etc.), smoking and alcohol consumption habits, diet, indoor air pollution, physical activity, female reproductive history, and sleep and mental statuses. The physical examination included measurements of height, weight, body fat, waist and hip circumference, electrocardiogram, and blood pressure, as well as the collection and storage of blood, urine, and mouthwash samples.

### 2.2 EBV serological antibody testing

The collected blood samples were centrifuged and transported by a cold chain to the central laboratory of the SYSUCC for testing. The levels of two biomarkers, EBNA1/IgA (Zhongshan Biotechnology, Zhongshan, China) and VCA/IgA (Euroimmun, Lübeck, Germany), were measured by enzyme-linked immunosorbent assay (ELISA) using commercial kits (24). The levels of these serum markers were determined using photometric methods following the manufacturer’s protocols. EBV antibody levels were standardized by the ratio of the optical density of the sample to the reference control (rOD). According to the ELISA kit standards, the criterion for positivity was rOD≥0.7 for EBNA1/IgA and rOD≥0.8 for VCA/IgA. The risk scores of the NPC were calculated using a risk prediction model: LogitPROB=−3.934+2.203×VCA/IgA+4.797×EBNA1/IgA (24). A predefined serological algorithm was used for risk stratification (low risk: PROB<0.65 ; medium risk: 0.65≤PROB<0.98 ; high risk: PROB≥0.98 ) (9, 25). Based on this algorithm, the samples classified as intermediate and high risk were considered positive for EBV reactivation, whereas those with low risk were judged to be negative for EBV reactivation.

### 2.3 Nested case–control study

We used a nested case–control study approach to improve the comparability of the data. The EBV reactivation status associated with NPC was used as the dependent variable, matched 1:1 by marriage and income. A total of 1596 pairs of participants were eventually included in the study. Furthermore, 70% of these data were used as a training set for the constructed model, and the remaining 30% were used as a test set to validate the performance of the constructed model. Finally, the training and test sets comprised 1124 and 472 pairs of participants, respectively.

### 2.4 Bayesian network

A BN is a DAG based on probabilistic inference, which consists of a set of nodes representing each variable and arcs showing the relationships between the nodes (26, 27). BN can represent the complex relationships of variables in a given problem in a network structure, reflecting the dependencies of variables in the problem domain through a network model suitable for representing and reasoning about uncertain knowledge. Because of its acyclic nature, the relationships between variables within a BN are often described by “family analogies,” where the nodes of interest may have parent and child nodes (28). When an arc extends from variable A to variable B (A → B), this indicates that variable A either has a direct influence on variable B or is a risk factor for it. In this context, variable A is referred to as the parent node of variable B, and variable B is considered the child node of variable A (20). BNs comprise two components: structure learning and parameter learning. Structure learning involves determining an appropriate BN topology using a training sample set and combining it with prior knowledge. Parameter learning entails determining the conditional probability densities at each node given the BN topology.

This study used the following structural learning algorithms to construct BNs and compared them: score-based TABU search algorithm (29), hybrid-based max-min hill-climbing (MMHC) algorithm (30), and combined TABU and MMHC algorithm. We also attempted to construct averaged models learned using the same algorithms but applying model averaging techniques over an ensemble of 5000 network structures so as to give the model better predictive performance and reduce overfitting (31). Hence, six BN structures were constructed in this study using the following algorithms: TABU, MMHC, TABU after model averaging (Avg.TABU), MMHC after model averaging (Avg.MMHC), TABU combined with MMHC algorithm (TABU + MMHC), and TABU after model averaging and MMHC after model averaging combined algorithm (Avg.TABU + MMHC). We used Bayesian posterior estimation for parameter learning of the BN.

### 2.5 Structural equation modeling

When using different BN algorithms to obtain the causal structure, SEM can stabilize the variability of the results and compare the received models to obtain a more reliable structure (32). SEM was applied in this study to select the most reliable structure among the BNs constructed by combining algorithms (TABU + MMHC and Avg.TABU + MMHC). An example of a BN constructed by combining the TABU and MMHC algorithms was given. An initial network structure was first constructed, including arcs that appeared in both structures. The initial structure was then fitted to the data using SEM. Next, uncertain arcs (e.g., arcs that appeared in the TABU-based model structure but not in the MMHC-based model structure) were sequentially added to the model, which was then compared using chi-square tests to retain the best structure for model fitting (33).

### 2.6 Definition of variables

The information of interest was collected through questionnaires, and the continuous variables were transformed into categorical variables such as age (≤40, 41–50, 51–60, and >60), body mass index (<18.5, 18.5–24.9, 25.0–29.9, and ≥30 kg/m^2^), and income (<50,000, 50,000–80,000, 80,000–100,000, and ≥100,000 ¥). Other categorical variables included sex (female and male), education level (primary school or less, secondary school, and college/university or more), marriage (married and unmarried), smoking (nonsmokers, former smokers, and current smokers), preserved foods (no and yes), cooking (no and yes), family history of cancer (no/yes), tea consumption (non-tea drinkers, occasional tea drinkers, and regular tea drinkers), soup consumption (no/yes), and herbal tea consumption (no/yes). Current smokers were defined as those who had smoked 100 cigarettes in their lifetime and were still smoking, former smokers as those who had quit smoking for more than 6 months, and nonsmokers as those who did not meet these criteria. Regular tea drinkers were defined as those who drank tea at least 1 day a week, occasional tea drinkers as those who drank tea at most one to three times a month, and non-tea drinkers as those who did not fulfill these criteria.

### 2.7 Statistical analysis

The categorical variables were expressed as percentages. We used univariate logistic regression to initially analyze the potential risk factors for EBV reactivation and multivariate stepwise logistic regression analyses for variables that were statistically significant as independent variables. All statistical analyses were performed using R software (version 4.2.2, R Core Team, Vienna, Austria). *P* values <0.05 indicated statistically significant differences. BN structure learning was implemented using the “bnlearn” package (34) in R software, and SEM was implemented using the “lavaan” package (33). The tenfold cross-validation was applied to evaluate the prediction performance of BN models constructed using different structural algorithms. Bayesian posterior estimation was used to learn the parameters of the BN model. This study also used Netica software (version 6.09, Norsys Software Corp., BC, Canada) to visualize the BN model and BN inference. Finally, we compared the predictive performance of the logistic model with that of the BN model using confusion matrix calculations.

## 3 Results

### 3.1 Baseline characteristics of the study population

**Figure 1:**
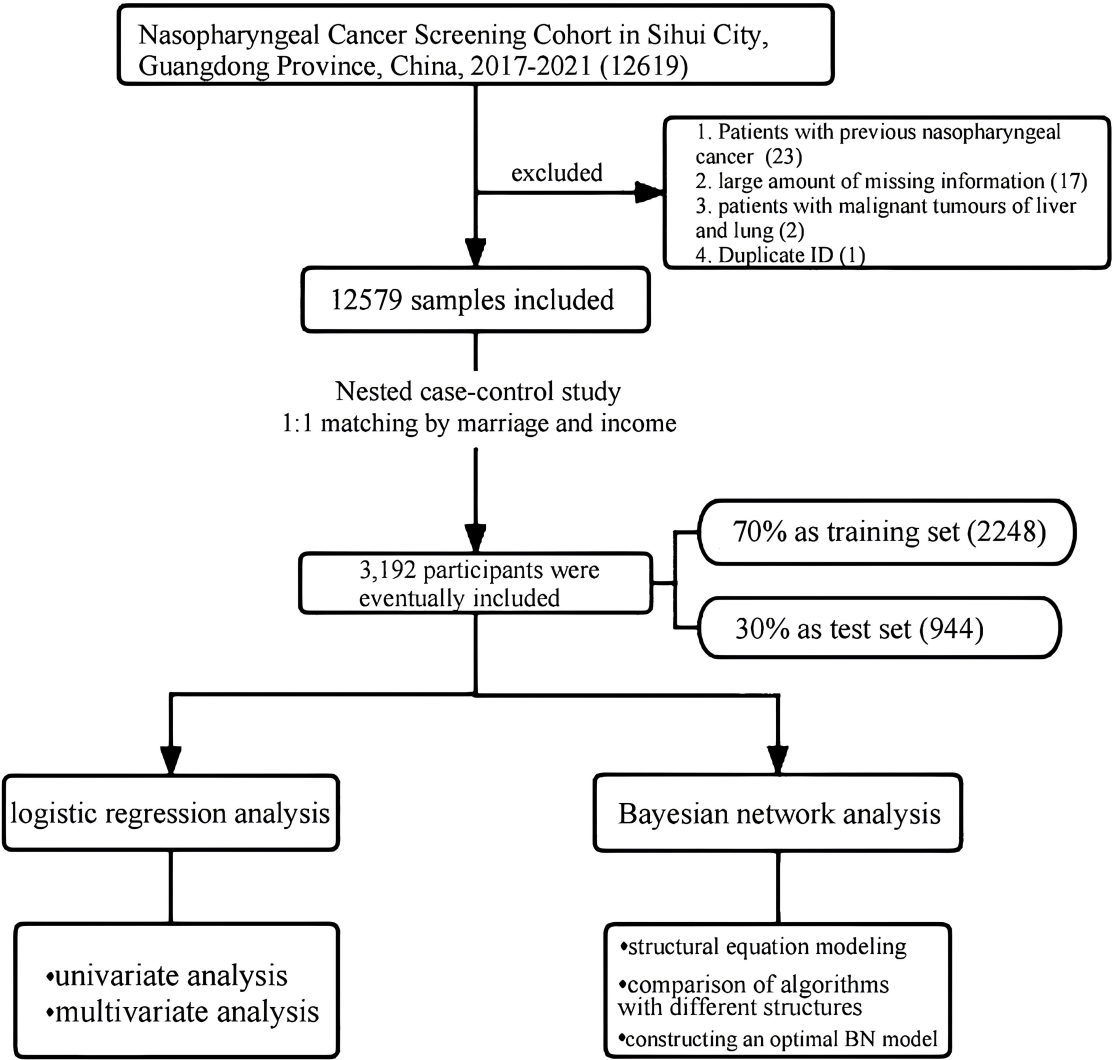
The screening process for research samples.

In this study, 23 patients with NPC previously, 17 participants with missing essential information, 2 patients with liver and lung malignancies, and 1 participant with duplicate information were excluded, leaving 12,579 participants in the analysis. The sample screening flowchart is shown in **Figure 1**. The baseline characteristics of the study participants are depicted in **Table 1**. The mean age of the participants was 51.67 ± 9.52 years (range: 20–77 years). More female patients were recruited into the study, with a male-to-female sex ratio of approximately 2:5 (3724:8855). Most participants (92.14%) were married, and more than half (54.29%) had completed secondary education. Further, 65.82% of the participants were of normal weight, 27.03% were overweight, and almost 30% (30.55%) had a family history of cancer. Most participants were in the habit of drinking soup (92.35%) and herbal tea (71.47%).

**Table 1:** Characteristics of the participants in this study, conducted in Sihui City, Guangdong Province, China, during 2017–2021 (*n* = 12,579).

Characteristic	Research objects (*n* = 12579)
**Age, y**( $\bar{X}\pm S$ )	51.67 ± 9.52
Age group(*n*,%)	
≤40	1650(13.12)
41-	4250(33.79)
51-	4103(32.62)
>60	2576(20.48)
Sex(*n*,%)	
Female	8855(70.40)
Male	3724(29.60)
Class_P(*n*,%)	
Low-risk	10965(87.17)
Medium-risk	1196(9.51)
High-risk	418(3.32)
Education level(*n*,%)	
Primary school or less	3703(29.44)
Secondary school	6829(54.29)
College/University or more	2042(16.23)
Others	5(0.04)
Marital status(*n*,%)	
Married	11590(92.14)
Unmarried	974(7.74)
Others	15(0.12)
Cigarette smoking(*n*,%)	
Never	10419(82.83)
Current	1741(13.84)
Former	411(3.27)
Others	8(0.06)
Preserved food(*n*,%)	
No	8025(63.80)
Yes	4548(36.16)
Others	6(0.05)
Cooking(*n*,%)	
No	1503(11.95)
Yes	11055(87.88)
Others	21(0.17)
Income(*n*,%)	
<50,000	2633(20.93)
50,000-80,000	3266(25.96)
80,000-100,000	3002(23.87)
≥100,000	3664(29.13)
Others	14(0.11)
Family history of cancer(*n*,%)	
No	8299(65.98)
Yes	3843(30.55)
Others	437(3.47)
Tea drinking(*n*,%)	
Never	5776(45.92)
Former	4386(34.87)
Current	2405(19.12)
Others	12(0.10)
Herbal tea drinking(*n*,%)	
No	3567(28.36)
Yes	8990(71.47)
Others	22(0.17)
Soup drinking(*n*,%)	
No	933(7.42)
Yes	11617(92.35)
Others	29(0.23)

Based on the EBV reactivation status, the study participants were matched 1:1 by marriage and income, including 1124 EBV reactivation-positive and 1124 EBV reactivation-negative patients in the training set. **Table 2** presents the baseline characteristics of the study participants after matching. The EBV reactivation-positive group had a higher percentage of individuals who were 60 years of age or older, were male, had a primary school education or less, and were both former and current smokers compared with the EBV reactivation-negative group.

**Table 2:** Characteristics of the participants after matching (*n* = 2248).

Characteristic	EBV reactivation status	
	Negative(*n* = 1124)	Positive(*n* = 1124)
Age group(*n*,%)		
≤40	144(12.81)	115(10.23)
41-	366(32.56)	312(27.76)
51-	377(33.54)	362(32.21)
>60	237(21.09)	335(29.80)
Sex(*n*,%)		
Female	809(71.98)	732(65.12)
Male	315(28.02)	392(34.88)
Education level(*n*,%)		
Primary school or less	347(30.87)	381(33.90)
Secondary school	597(53.11)	606(53.91)
College/University or more	180(16.01)	137(12.19)
Cigarette smoking(*n*,%)		
Never	942(83.81)	869(77.31)
Current	143(12.72)	207(18.42)
Former	39(3.47)	48(4.27)
Preserved food(*n*,%)		
No	712(63.35)	747(66.46)
Yes	412(36.65)	377(33.54)
Cooking(*n*,%)		
No	113(10.05)	145(12.90)
Yes	1011(89.95)	979(87.10)
Family history of cancer(*n*,%)		
No	788(70.11)	791(70.37)
Yes	336(29.89)	333(29.63)
Tea drinking(*n*,%)		
Never	536(47.69)	500(44.48)
Former	379(33.72)	374(33.27)
Current	209(18.59)	250(22.24)
Herbal tea drinking(*n*,%)		
No	332(29.54)	315(28.02)
Yes	792(70.46)	809(71.98)
Soup drinking(*n*,%)		
No	91(8.10)	75(6.67)
Yes	1033(91.90)	1049(93.33)
Marital status(*n*,%)		
Married	1031(91.73)	1031(91.73)
Unmarried	93(8.27)	93(8.27)
Income(*n*,%)		
<50,000	252(22.42)	252(22.42)
50,000-80,000	324(28.83)	324(28.83)
80,000-100,000	257(22.86)	257(22.86)
≥100,000	291(25.89)	291(25.89)

### 3.2 Univariate analysis

We first performed univariate logistic regression analysis to investigate the potential risk factors for EBV reactivation. The results showed that consuming preserved food, having a family history of cancer, and drinking herbal tea and soup had no statistically significant effect on EBV reactivation (*P* > 0.05). However, sex (OR = 1.375, 95% CI *=* 1.150–1.645 for men), age (OR = 1.770, 95% CI *=* 1.316–2.380 for age more than 60 years), education level (OR = 0.693, 95% CI *=* 0.532–0.904 for college/university or more), smoking status (OR = 1.569, 95% CI *=* 1.244–1.979 for current smokers), cooking (OR = 0.755, 95% CI *=* 0.581–0.980), and tea drinking (OR = 1.282, 95% CI *=* 1.029–1.599 for regular tea drinkers) had a statistically significant effect on EBV reactivation (*P* < 0.05) (**Table 3**).

**Table 3:** Influencing factors of EBV reactivation status analyzed using univariate logistic regression (*n* = 2248).

Variable	OR(95% CI)	*P*
Sex		
Female	1.000(reference)	
Male	**1.375(1.150,1.645)**	**<0.001**
Age, y		
≤40	1.000(reference)	
41-	1.067(0.800,1.424)	0.657
51-	1.202(0.905,1.598)	0.204
>60	**1.770(1.316,2.380)**	**<0.001**
Education level		
Primary school or less	1.000(reference)	
Secondary school	0.924(0.769,1.111)	0.403
College/University or more	**0.693(0.532,0.904)**	**0.007**
Cigarette smoking		
Never	1.000(reference)	
Current	**1.569(1.244,1.979)**	**<0.001**
Former	1.334(0.866,2.056)	0.191
Preserved food		
No	1.000(reference)	
Yes	0.872(0.733,1.037)	0.122
Cooking		
No	1.000(reference)	
Yes	**0.755(0.581,0.980)**	**0.035**
Family history of cancer		
No	1.000(reference)	
Yes	0.987(0.824,1.183)	0.890
Tea drinking		
Never	1.000(reference)	
Former	1.058(0.877,1.276)	0.557
Current	**1.282(1.029,1.599)**	**0.027**
Herbal tea drinking		
No	1.000(reference)	
Yes	1.077(0.897,1.292)	0.428
Soup drinking		
No	1.000(reference)	
Yes	1.232(0.897,1.693)	0.198

Bold means that sex, age, education level, smoking status, cooking, and tea drinking had a statistically significant effect on EBV reactivation (*P* < 0.05).

### 3.3 Multivariate analysis

In this study, a multivariate logistic regression analysis of the factors influencing EBV reactivation was performed using a stepwise approach to construct a logistic regression model with EBV reactivation status as the dependent variable and the variables significantly associated with EBV reactivation as independent variables in the univariate logistic regression analysis (**Table 4**). The final results showed that only being older than 60 years (OR = 1.718, 95% CI *=* 1.273–2.322) and being a current smoker (OR = 1.477, 95% CI *=* 1.167–1.872) were the risk factors for EBV reactivation. This was consistent with a previous study (10).

**Table 4:** Influencing factors of EBV reactivation status analyzed using multivariate logistic regression (*n* = 2248).

Variable	Estimate	*S.E*	*P*	OR(95% CI)
(Intercept)	-0.081	0.173	0.638	0.922(0.657,1.293)
Age of 41 to 50	0.067	0.148	0.650	1.069(0.801,1.429)
Age of 41 to 50	0.161	0.146	0.270	1.175(0.883,1.566)
Age over 60	0.541	0.153	**<0.001**	**1.718(1.273,2.322)**
Current-smoker	0.390	0.120	**<0.001**	**1.477(1.167,1.872)**
Former-smoker	0.083	0.227	0.713	1.087(0.698,1.701)
Cooking	-0.217	0.136	0.111	0.805(0.615,1.051)

Bold means that being older than 60 years and being a current smoker had a statistically significant effect on EBV reactivation (*P* < 0.05).

### 3.4 Bayesian network model

In the present study, six BN structures were constructed using different learning algorithms and then compared. The results are presented in **Table 5**. The combination of the average TABU and MMHC structures balanced by SEM was better on all dimensions of assessment compared with the performance of the other structures, achieving the highest recall (i.e., 0.5391) and the lowest prediction error (0.4558). This suggested that balanced structures could be found to provide models with high predictive performance. Consequently, the final choice in this study was to construct the BN with balanced mean TABU and MMHC structures.

**Table 5:** Results of cross-validation using different BN structures.

Algorithm	TABU	MMHC	Avg. TABU	Avg. MMHC	TABU+ MMHC	Avg.TABU+ MMHC
Sensitivity	0.5238	0.4930	0.4857	0.4826	0.5365	0.5391
Specificity	0.5355	0.4928	0.4848	0.4807	0.5568	0.5658
Precision	0.6269	0.5004	0.5004	0.5080	0.6485	0.6765
Recall	0.5238	0.4930	0.4857	0.4826	0.5365	0.5391
F1	0.5707	0.4967	0.4930	0.4950	0.5872	0.6000
Prevalence	0.5984	0.5076	0.5151	0.5263	0.6038	0.6275
Detection Rate	0.3134	0.2502	0.2502	0.2540	0.3240	0.3382
Detection Prevalence	0.5000	0.5000	0.5000	0.5000	0.5000	0.5000
Balanced Accuracy	0.5296	0.4929	0.4853	0.4817	0.5467	0.5524
BIC loss (SD)	7.1477 (0.0033)	7.1527 (0.0022)	7.1521 (0.0029)	7.1372 (0.0023)	7.1492 (0.0018)	7.1319 (0.0033)
BDE loss (SD)	7.1472 (0.0034)	7.1521 (0.0041)	7.1513 (0.0030)	7.1372 (0.0020)	7.1499 (0.0015)	7.1340 (0.0014)
Prediction loss(SD)	0.4765 (0.0061)	0.5008 (0.0080)	0.5025 (0.0108)	0.5056 (0.0083)	0.4588 (0.0056)	0.4558 (0.0029)

**Figure 2:**
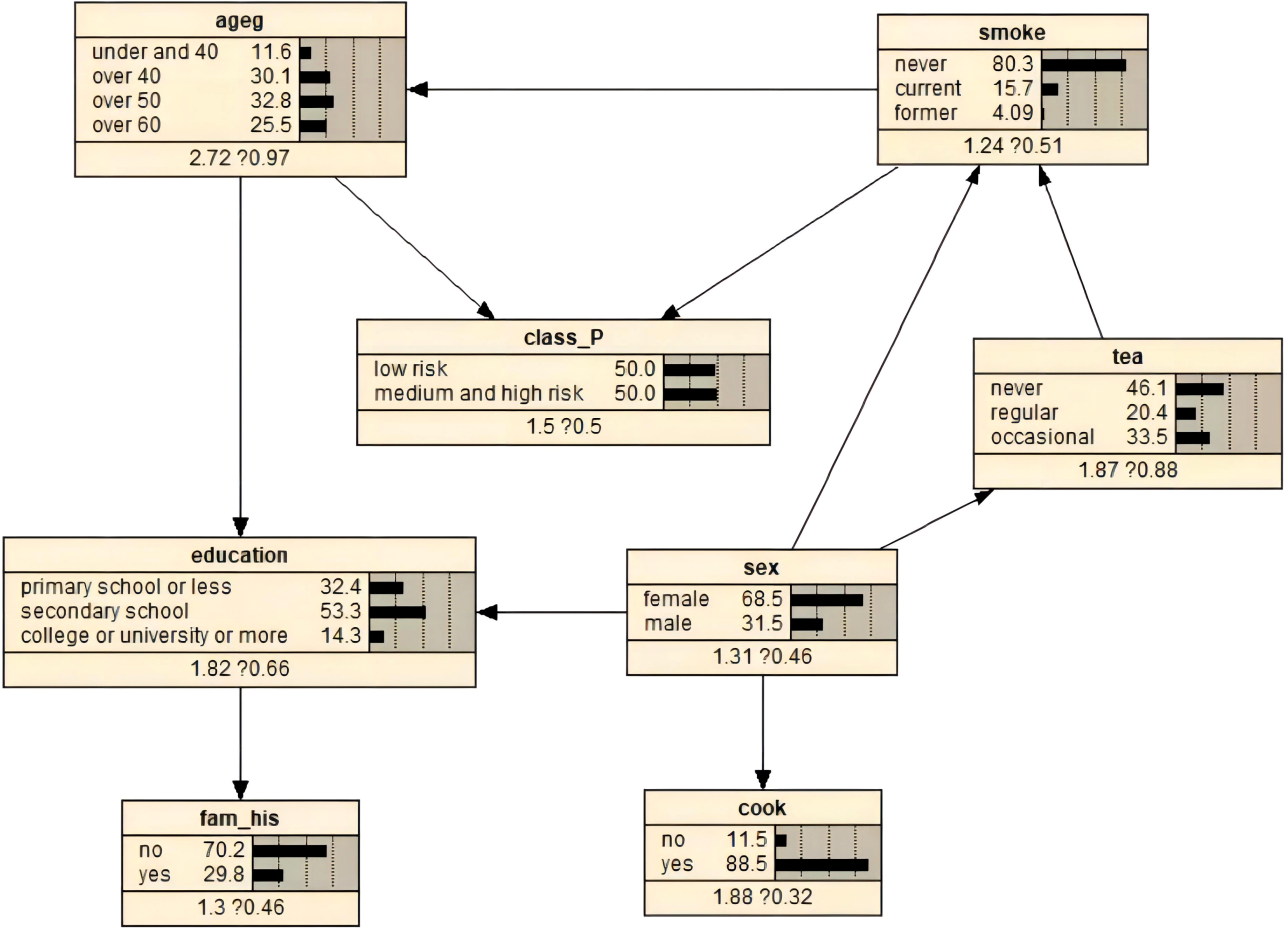
BNs and prior probability of EBV reactivation status constructed using the optimal algorithm.

**Figure 2** shows the optimal BN constructed from the balanced average TABU and MMHC structures. The network was constructed from 8 nodes and 10 directed edges, indicating the probabilistic dependencies between connected nodes. The results showed that age and smoking status were the parent nodes of class_P, that is, the direct influence on EBV reactivation. However, sex, education level, tea drinking, cooking, and family history of cancer were indirect influencing factors of EBV reactivation. In addition, the model showed that age and sex had a direct effect on education level and sex had a direct effect on cooking status. Furthermore, tea drinking was associated with sex and smoking status.

### 3.5 Bayesian inference

**Figure 3:**
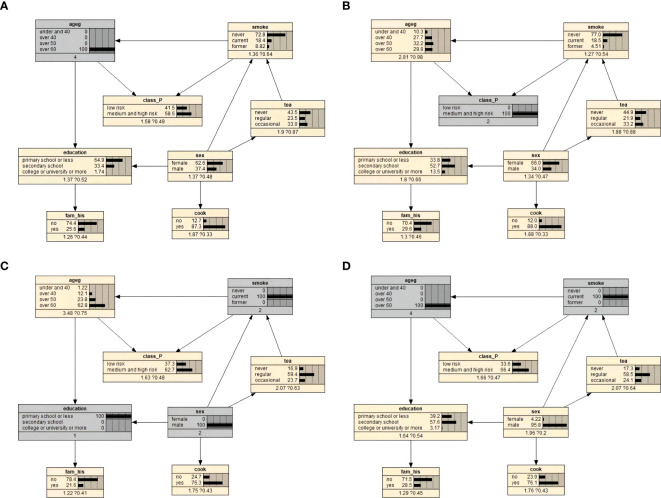
Bayesian network conditional probability inference results on EBV reactivation state. **(A)** Conditional probability when the individual was 60 years old and above. **(B)** Conditional probability when an individual was positive for EBV reactivation. **(C)** Conditional probability when an individual was over 60 years old and currently smoked. **(D)** Conditional probabilities for men who currently smoked and had primary school education or less.

The prior probabilities of the variables are illustrated in **Figure 2**. We used Bayesian posterior estimation to learn the parameters of the network model. The resulting probabilistic model was used to analyze the effect of these variables on EBV reactivation by calculating conditional probabilities. For example, if an individual was aged 60 years or older, the probability of EBV reactivation increased from 50% to 58.5% (**Figure 3A**). If a person was positive for EBV reactivation, the probability that their sex was male increased from 31.5% to 34.0%, and the probability that they had never smoked decreased from 80.3% to 77.0% (**Figure 3B**). If a person was older than 60 years and smoked, the probability of being EBV reactivation positive, that is classified as having a high risk of NPC, was 66.4% (**Figure 3C**). In contrast, a man who smoked and had a primary school education or less had a 62.7% chance of being EBV reactivation positive (**Figure 3D**).

## 4 Discussion

In previous studies, logistic regression was usually used to analyze NPC-associated risk factors for EBV reactivation, the results of which were better understood and more acceptable to researchers. However, the shortcomings of the regression model are magnified in a network framework (14). First, the assumption of linear additivity of the model may limit its use. Second, the performance of the regression model is more likely to be affected by covariance between variables in datasets with complex relationships. Third, the regression model cannot capture the complex network of interactions between multiple risk factors. Although this problem can be solved by adding interaction terms, the number of possible interactions increases exponentially with the increase in the number of variables, leading to a complex interaction process and thus reducing the effectiveness of the model test. However, the BN model can compensate for the aforementioned deficiencies of the regression model. As a result, BNs have been increasingly favored by clinical researchers in recent years as a risk assessment tool for large clinical datasets.

The BN model suggests that EBV may be reactivated due to poor daily living habits, unhealthy cooking environments, decreased host immunity, and having specific genes. Smoking directly exposes the nasopharyngeal epithelium to tobacco smoke. It has been suggested that smoking may promote nasopharyngeal carcinogenesis not only through the direct carcinogenic effect of tobacco smoke, but also indirectly by inducing EBV reactivation (35, 36). The rationale may be that exposure to nicotine promotes NPC cell proliferation and EBV replication and expression of its cleaved gene products (37). Similarly, in a prospective screening study based in southern China, Hu et al. (38) found that smoking was associated with EBV serum (VCA/IgA, EBNA1/IgA) positivity during a 3- to 5-year follow-up period, and that participants aged 60 to 69 years had a higher risk of EBNA1/IgA positivity. In addition, some studies have found an increased risk of EBV reactivation in people who have used solid fuels continuously for more than 40 years compared with those who do not cook or use cleaner fuels, and that ingestion of preserved foods may exacerbate the effect of solid fuels on the risk of EBV reactivation (39). During the combustion of solid fuels, incomplete combustion results in the emission of complex mixtures of gaseous and particulate pollutants; these products, such as polycyclic aromatic hydrocarbons (PAHs), have been found to cause immune damage (40, 41) and lead to changes in the reactivation of defense viruses (42). The nasopharynx is the first site of deposition of solid fuel combustion products, and the pro-inflammatory effect on epithelial cells may disrupt the inflammatory balance and affect the bacterial and viral flora of the nasopharynx (43).

This study also visually reasoned the constructed BN model using Netica software. Moreover, we compared the predictive performance of the two models using the test set. The results are depicted in **Table 6**. The table demonstrates that the accuracy (56.36%) was consistent. The BN slightly outperformed the traditional logistic regression model in all indicators. The BN, being based on the interaction network, not only predicted the direct or indirect risk factors of the disease but also facilitated visual inference. Therefore, the BN model might be considered a novel predictive model in studying NPC-related risk factors for EBV reactivation.

**Table 6:** Evaluation indices of logistic regression and Bayesian network models.

Model type	Accuracy (%)	Precision (%)	Sensitivity (%)	Specificity (%)	F1 (%)	AUC
Logistic regression	56.36	57.18	43.22	69.49	49.23	0.5520
BN	56.36	58.52	43.64	69.07	50.00	0.5669

NPC is a complex disease whose development is associated with genetic susceptibility, EBV infection, and environmental factors (44–46). Non-keratinizing NPC is the main type of NPC in China, and almost all patients with non-keratinizing NPC have EBV infection. Also, many other factors affect EBV reactivation. However, based on the South China Chronic Disease Cohort Study, we only considered the influence of the living habits of people in high-prevalence areas on EBV reactivation. Besides, the selection of different EBV-related markers might also have influenced the results. Under the hypothesis that antibodies against the remaining EBV proteins may perform better than those already reported, Li et al. (47) designed a peptide library containing highly sorted B-cell epitopes of EBV based on linear B-cell epitope prediction. The serum antibodies, including IgA, IgG, and Ab, to the peptide fragment P85 expressed by the *BNLF2b* gene differed significantly between cases and controls, with P85-Ab having the best performance (area under the curve = 0.97). This novel NPC screening marker might substantially improve the performance of screening methods for NPC populations and increase the cost-effectiveness of screening, thus shedding new light on our study of the risk factors for NPC-associated EBV reactivation.

To consider intricate interactions between factors, this study applied BN to analyze the risk factors associated with EBV reactivation in NPC and used SEM tools in constructing BN. The results of the study not only revealed the risk factors for EBV reactivation associated with NPC but also identified the direct and indirect influences on EBV reactivation and elucidated their complex interactive network relationships. In addition, the model, with Bayesian visualization of predictive inference, can raise our awareness of nasopharyngeal cancer prevention in our daily life, suggesting that we should maintain good living habits, such as quitting smoking, not staying up late, using ventilated and clean cooking environments, and exercising. This not only has a positive effect on the middle- and high-risk groups to actively participate in follow-up screening, but also helps in clinical decision-making, which can improve the early detection rate of nasopharyngeal cancer, which is of great significance to the prognosis of nasopharyngeal cancer patients, and provides a new way of thinking about the prevention of nasopharyngeal cancer. However, this study had some limitations. First, some of the data in this study were collected through structured questionnaires, and participants are also required to review their lifestyle habits from the past six months or even several years ago, such as eating pickled foods, smoking and drinking. So recall bias was inevitable. Second, the directed edges in BNs did not indicate causal relationships between connected nodes. Instead, they represented probabilistic dependencies. Third, most of the invalid variables were found in the construction of the logistic regression model, which could be partly explained by undifferentiated misclassification. In addition, we did not find an association between the consumption of salty food and EBV reactivation. This lack of association could be attributed to the extended duration of the NPC screening study in Sihui City, leading to a gradual shift in local residents’ dietary habits. Fourth, this study was only based on the investigation in Sihui City, Guangdong Province, and the extrapolation of data was affected. Fifth, this study did not attempt to use other antibody biomarkers of EBV reactivation for comparison.

EBV reactivation is also associated with a number of diseases. The present study is only a preliminary study of the relationship between lifestyle habits and EBV reactivation, and subsequent studies could further incorporate a number of chronic non-communicable diseases (e.g., hypertension, diabetes mellitus, stroke, etc.) to explore the relationship between them and EBV reactivation. Moreover, We plan to conduct multicenter studies with more EBV reactivation antibody biomarkers and more variable parameters in the future to construct more accurate and reliable BN models of EBV reactivation risk factors associated with NPC. And we will try to construct BN prediction models for other diseases in order to build a more mature and stable prediction network to provide methodological references for the prevention of more diseases.

## 5 Conclusions

This study demonstrated that BN based on SEM balanced mean TABU and MMHC combined algorithm could not only realize the complex network relationship between risk factors and NPC-associated EBV reactivation but also made it possible to predict the risk of EBV reactivation. The findings provided a scientific idea for preventing and treating NPC, contributing to the reduction of its prevalence. The specific findings were as follows: (1) The logistic regression model showed that NPC-related EBV reactivation was significantly associated with age and smoking status, whereas stable environmental factors had no association, suggesting that environmental factors might in turn influence NPC through other mechanisms. (2)The BN model of EBV reactivation was constructed using 8 nodes and 10 directed edges. Age and smoking status were the direct influencing factors for EBV reactivation. In contrast, sex, education level, tea drinking, cooking, and family history of cancer constituted the indirect influencing factors of EBV reactivation. (3) The BN using the SEM balanced average TABU and MMHC combined algorithm could achieve the probabilistic inference of unknown nodes through known nodes and flexibly demonstrated the influence of a risk factor on EBV reactivation. (4) Overall, the BN with SEM balanced average TABU and MMHC combined algorithm could be used as a new model to predict risk factors for NPC-associated EBV reactivation, with a broad prospect in the clinical practice of NPC.

## Data Availability

The datasets presented in this article are not readily available because confidentiality agreement for data use. Requests to access the datasets should be directed to Director Cao Sumei of the Sun Yat - sen University Cancer Center, caosm@sysucc.org.cn.
